# Sea-Level Rise Induced Multi-Mechanism Flooding and Contribution to Urban Infrastructure Failure

**DOI:** 10.1038/s41598-020-60762-4

**Published:** 2020-03-02

**Authors:** Shellie Habel, Charles H. Fletcher, Tiffany R. Anderson, Philip R. Thompson

**Affiliations:** 1 University of Hawai’i at Mānoa, School of Ocean and Earth Science and Technology, Department of Earth Sciences, POST Building, Suite 701, 1680 East-West Road, Honolulu, HI 96822 USA; 2 University of Hawai’i at Mānoa, Sea Level Center, 1000 Pope Road, MSB 317, Honolulu, HI 96822 USA

**Keywords:** Climate-change adaptation, Climate-change impacts

## Abstract

Sea-level rise (SLR) induced flooding is often envisioned as solely originating from a direct marine source. This results in alternate sources such as groundwater inundation and storm-drain backflow being overlooked in studies that inform planning. Here a method is developed that identifies flooding extents and infrastructure vulnerabilities that are likely to result from alternate flood sources over coming decades. The method includes simulation of flood scenarios consisting of high-resolution raster datasets featuring flood-water depth generated by three mechanisms: (1) direct marine flooding, (2) storm-drain backflow, and (3) groundwater inundation. We apply the method to Honolulu’s primary urban center based on its high density of vulnerable assets and present-day tidal flooding issues. Annual exceedance frequencies of simulated flood thresholds are established using a statistical model that considers predicted tide and projections of SLR. Through assessment of multi-mechanism flooding, we find that approaching decades will likely feature large and increasing percentages of flooded area impacted simultaneously by the three flood mechanisms, in which groundwater inundation and direct marine flooding represent the most and least substantial single-mechanism flood source, respectively. These results illustrate the need to reevaluate main sources of SLR induced flooding to promote the development of effective flood management strategies.

## Introduction

The City and County of Honolulu and the State of Hawai’i as a whole are part of a global consortium of forward-thinking coastal regions working to develop solutions to sea-level rise (SLR) related challenges. In recent years, reports have been issued by local government that highlight the increasing vulnerability of urbanized areas to SLR induced flooding^1,2^. Such efforts are crucial towards the design of flood management strategies; however, the dismissal of main sources of SLR related flooding can potentially render management strategies ineffective.

Among the State of Hawai’i’s municipalities, Honolulu represents the highest potential for SLR related economic losses^3^. Much of this vulnerability stems from limited tidal ranges that have allowed a dense network of development to take place in close proximity to the shoreline and has also resulted in Honolulu having some of the lowest elevation flood thresholds in the United States^4^, similar to Baltimore, Washington D.C., San Francisco, and others^5^. Further, the area hosts a shallow grade such that small increments of SLR is expected to cause extensive lateral shifts in impacted area^6^.

**Figure 1:**
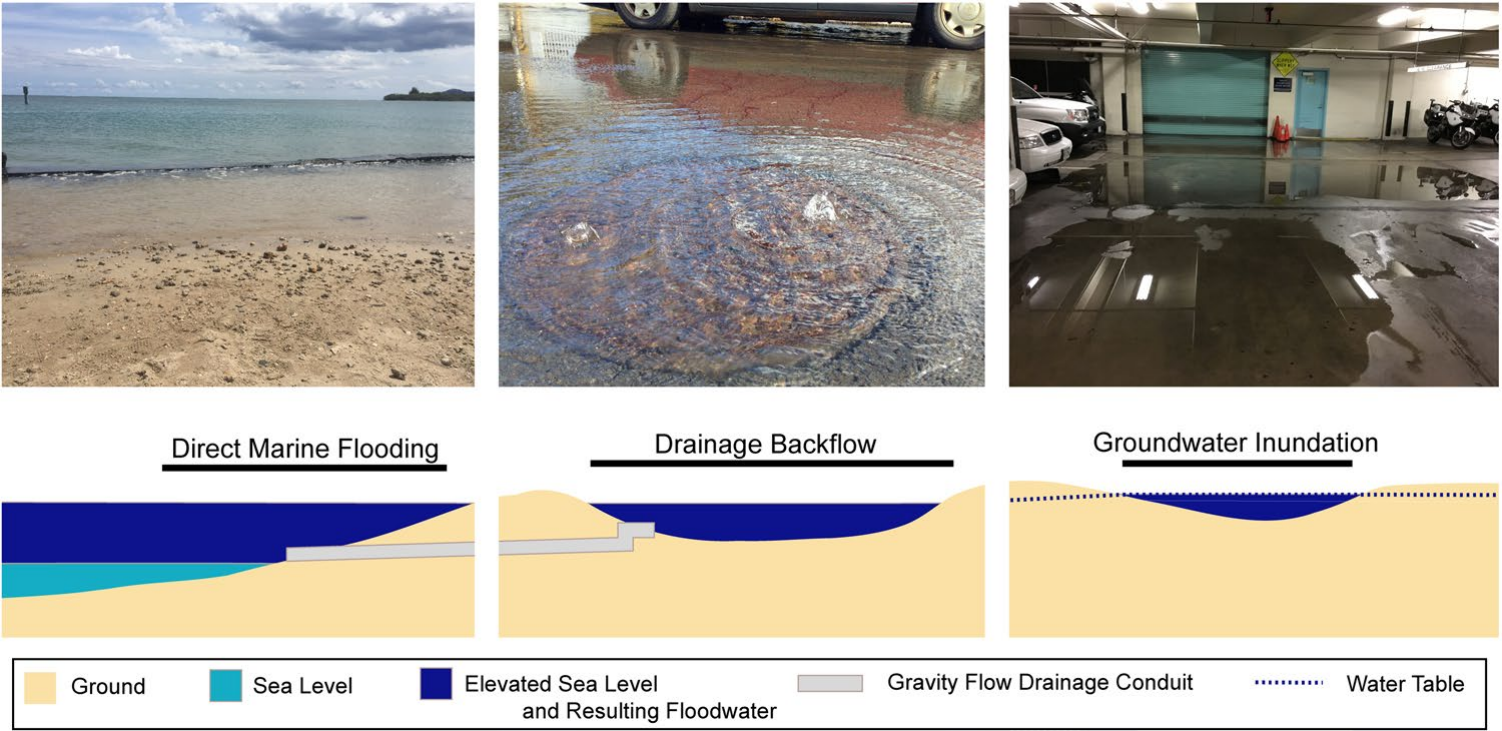
Observations and illustrations of direct marine flooding, storm-drain backflow, and groundwater inundation within Honolulu. Each mechanism of flooding has already been observed during periods of extreme tide in various locations within the study area. Photo Credit, Hawai’i and Pacific Islands King Tide Project^17^.

Three main types of flooding have become problematic within the Honolulu’s urban center during extreme high tide events (Fig. 1)^1^. The most notable events were observed during the summer of 2017 when a combination of anomalously high mean sea levels and seasonally high tides contributed to record water levels reaching more than 0.35 m above the mean higher high water (MHHW) datum at the Honolulu Tide Station^7,8^. During these events, several types of infrastructure impacts were observed: roadway near the Honolulu International Airport was inundated by direct marine flooding; GWI impacted basements in urban Honolulu; and storm-drain backflow produced flooding in underground parking garages and contributed to traffic congestion in parts of Waikīkī. Impacts resulting from the 2017 event, while relatively mild in comparison to those featured in other urban settings (i.e., Miami, Norfolk, Boston, New York, etc.) revealed the advent of infrastructure failure within Honolulu’s primary urban center. Ongoing SLR will undoubtedly exacerbate flooding from these three flood mechanisms and, because each type infiltrates through unique flood pathways, will likely require unique engineering strategies to manage unless they are managed simultaneously (i.e., vertical retreat). The three mechanisms and examples of mechanism-specific adaptation measures are described as follows:

- Direct marine inundation describes flooding that occurs by direct surficial connection to marine waters. In mapping studies, direct marine inundation is often characterized through identification of locations featuring topographic elevations below that of a given flood threshold in which marine waters can move unimpeded over the land surface^9,10^. Prevention of such flooding generally requires construction of water-tight continuous structures, like those implemented in New Orleans and the Netherlands^11–14^.
- Storm-drain backflow is like direct marine flooding in that floodwaters generally originate from a marine source; however, storm-drain backflow is facilitated by the presence of gravity-flow drainage networks. The use of such drainage networks is widespread among the world’s coastal municipalities^15^. Functionality of gravity-flow drainage depends partially on elevation differences between drainage and receiving outflow areas (i.e., ocean waters). In low-lying coastal areas, high tides decrease these elevation differences and can slow or reverse the rate of drainage^16^. During extreme tide events in Honolulu, reversal of flow has impacted basements and low-lying streets^17^. Management solutions in Honolulu have been limited; however, other municipalities have employed check-valves and pumps that transport water to higher elevation discharge areas (i.e., forced drainage)^11,18^.
- Groundwater inundation (GWI) describes flooding that occurs as groundwater is lifted above the elevation of the ground surface and/or buried infrastructure. It has been identified as one of the more difficult flood mechanisms to manage owing to its ability to evade coastal barriers designed to mitigate direct marine flooding^19^. Among much of Honolulu’s low-lying coastal areas, groundwater is already very near to the ground surface such that GWI is likely impacting submerged infrastructure at present (i.e., cesspools, basements, and others). This mechanism is expected to produce increased flooding relative to the direct marine source due to coastal groundwater levels being generally elevated relative to local mean sea level (LMSL) and directly influenced by sea-level fluctuations such as tides, wave set-up, and longer period sea-level variations^19–24^. In locations such as the Netherlands and New Orleans, GWI is mitigated through groundwater extraction^13,14^. However, responding to GWI in this manner has been found to cause subsidence and increase susceptibility to flooding, especially in areas of unconsolidated sediment^13,25^. Resulting subsidence can produce ground fractures and structural damage to various types of infrastructure, including buildings, roads, flood-control structures, drainage networks, electric and gas substations, and sewer lines^26^.

The Hawaiian Islands, and more specifically the primary urban center of Honolulu, have been the subject of pertinent studies regarding SLR induced flooding, the results of which are currently being used to inform policy and planning regarding SLR adaptation^3,7,19,24,27,28^. However, specific simulation of the three identified mechanisms of SLR induced flooding has not yet been accomplished.

A statewide assessment of SLR impacts was conducted in 2017 that entailed simulation of dynamical physical processes including erosion and wave run-up^3,28^. These simulations supplemented the widely employed “bathtub” approach that simulates passive flooding by identifying elevations below that of projected sea level^9,10^. The statewide assessment employed the bathtub approach to implicitly represent flooding from a combination of phenomena including passive direct marine inundation, drainage backflow, and GWI; however, the three mechanisms were not uniquely simulated.

Simulations of GWI were produced as part of two studies undertaken in Honolulu. The first study introduced the concept of GWI using a 1D analytical model^19^ and the second expanded upon the method using a 3D numerical modeling approach^24^. Both studies employed coastal groundwater level data for calibration and measurement of tidal influence, and further combined simulated water levels with a digital-elevation model (DEM) to assess GWI related vulnerabilities in Honolulu’s primary urban center.

### Study objectives

The objective of the present study is to provide simulations of multiple SLR induced flood mechanisms and related infrastructure vulnerabilities. These are produced for Honolulu’s primary urban center for proof of concept. Simulations are produced by uniquely simulating flood locations and depths generated by each of the three mechanisms and by overlapping flood simulations to identify areas susceptible to multi-mechanism flooding. They can be produced to represent generally any projected magnitude of SLR; however, for the purpose of this study flood simulations are generated to represent four established flood thresholds. These include three thresholds expected to produce disruptive, damaging and destructive coastal flooding^5^, and a fourth threshold that was reached multiple times during king tide events in 2017 that produced disruptive flooding in Honolulu’s lowest lying developed area (i.e., M$\bar{{\rm{a}}}$punapuna)^7^. Hereafter, these established flood thresholds are referred to as minor, moderate, major, and KT2017 thresholds, respectively.

Results of a statistical model are used to provide frequencies at which sea level will exceed given thresholds in Honolulu over approaching decades^7^. The statistical model considers secular local mean sea-level (LMSL) rise, annual LMSL variability, and the annual 99th percentile of astronomical tidal height. The model has previously been used to estimate when flooding is expected to progress from intermittent to chronic in which chronic flooding is defined as the point in which a defined flood threshold is exceeded more than 50 days per year for 9 in 10 years. Results of the model indicate the potential for rapid transitions in threshold exceedance in which the transition from intermittent to chronic can occur within as little as a decade. Projections of exceedance frequencies can be combined with simulations of SLR induced flooding to provide a powerful tool for agencies responsible for adaptation planning^29–31^.

Here we do not consider dynamic coastal processes such as coastal erosion or wave run-up, nor changes in land cover. Honolulu’s coastline within the primary urban center is unique relative to the more natural coastlines featured among the Hawaiian Islands in that it is heavily developed and hardened such that erosion will likely be managed by continued hardening. Further, we acknowledge that this study does not include simulation of rainfall induced flooding.

### Assessment of infrastructure failure

**Figure 2:**
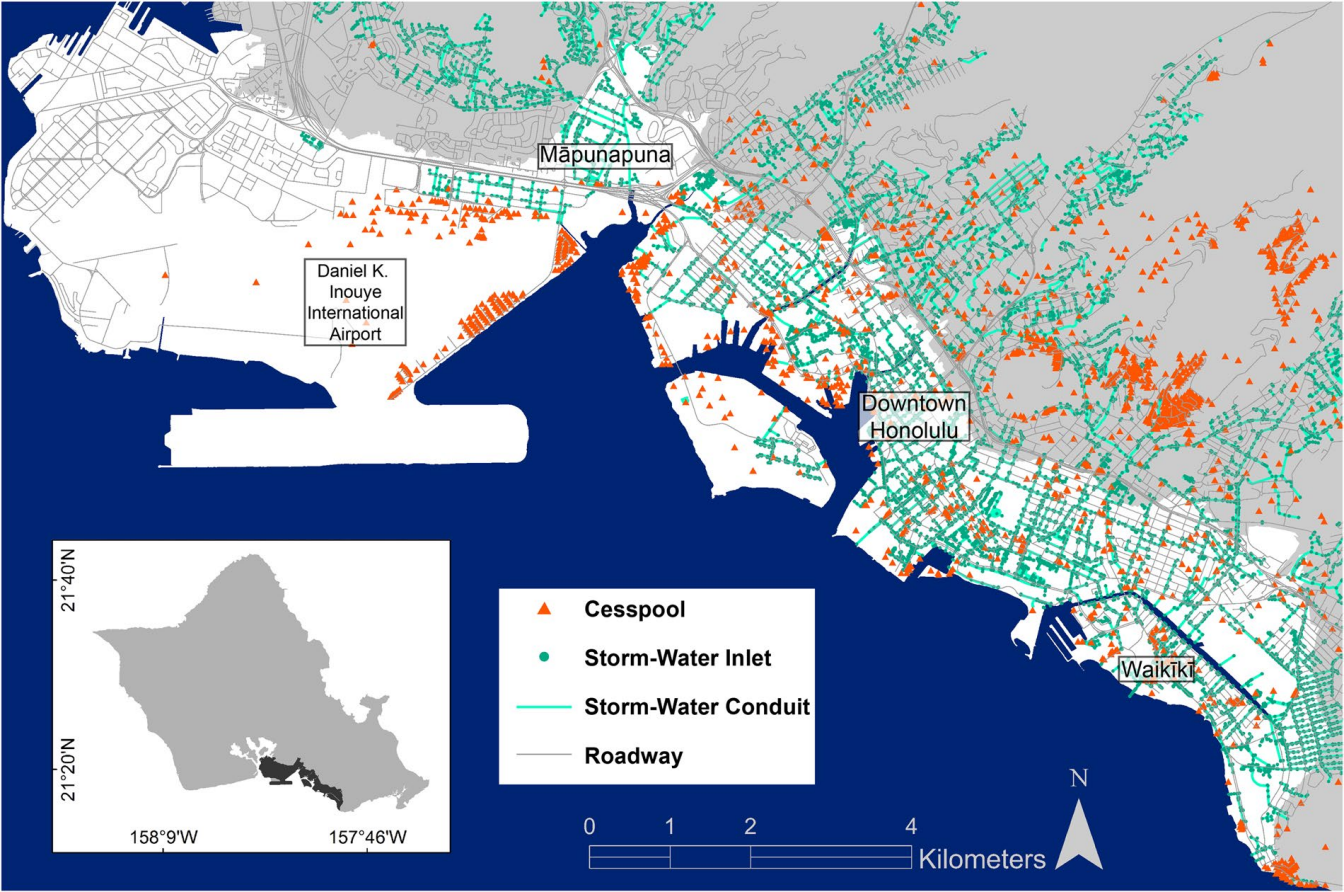
Honolulu’s Cesspool, Storm-Water Drainage, and Roadway Infrastructure. Study area extent shown in white. Infrastructure locations including cesspools, storm-water drainage conduits and inlets, and roadways are shown.

Using generated flood simulations, we assess critical infrastructure likely to fail such that the failure will cause direct ramifications as sea level reaches the four established flood thresholds. The assessment identifies (1) length and location of roadway segments likely to feature dangerous or impassable driving conditions, (2) number and location of gravity-flow drainage inlets (out of 6770) likely to fail and/or act as conduits for additional flooding, and (3) number and location of active cesspools (out of 691) likely to become non-functional at filtering effluent and/or be fully flooded to the ground surface. The infrastructure components considered are widespread among Honolulu’s heavily densified primary urban center (Fig. 2).

The failure of cesspool systems is particularly concerning owing to the potential for coastal groundwater contamination down-gradient of cesspool sites and in nearshore waters^32^. As SLR continues, the expanding presence of contaminated groundwater at the ground surface (in the form of GWI) is likely to cause increased opportunity for public exposure to contaminants.

Transportation systems are the main support for safety, commerce and the economy^33^ and reduction in performance has been identified as having some of the most costly impacts to society^34,35^. Notable SLR induced impacts are already occurring in coastal areas nationwide in the form of road closures and roadway degradation^36^. Here we focus on roadway failure owing to water depth and inability of cars to traverse, although it is understood that damages such as rutting and potholing will likely result from the presence of shallow water tables^37^. Assessment of such damages is outside the scope of this study and is recommended for future work.

### Local tides and sea-level rise projections

Based on measurements at the Honolulu tide station from 1905 to 2015, the semi-diurnal tide is 0.58 m and the local rate of SLR is 1.41 +/− 0.21 mm/yr^8^. The local mean higher-high water datum is 0.33 m relative to local mean sea level (LMSL). Acceleration in the rate of local SLR is expected to occur, however, the magnitude and timing of acceleration remain uncertain.

Alterations in local tidal amplitudes occur in response to changes in relative sea level^38^. At 92% of tide gauges located in the Pacific, sea level variability has been found to correlate with inter-annual tidal variability ranging from 1 to 50 cm per meter of SLR^39^. At the Honolulu tide gauge specifically, the added contribution of non-stationary tides to water levels is approximately 10% compared to that considering SLR and stationary tides^38,40^.

The most useful projections of SLR for local planning efforts account for changes in LMSL and forecasted local high tide flooding. The National Oceanic and Atmospheric Administration (NOAA) published a set of SLR scenarios reported by Sweet *et al*. that represent the secular component of sea level change. The study by Sweet *et al*. provided six scenarios of SLR discretized by 0.5-m increments that are associated with emissions based, conditional probabilistic scenarios and global model projections^4^. In this study we consider three local SLR projections provided by Sweet *et al*., characterized as the intermediate-low, intermediate, and intermediate-high scenarios. Each assume a non-linear rate of rise in which the intermediate-low scenario reaches 0.25 and 0.53 m in Honolulu by 2050 and 2100, respectively, the intermediate scenario reaches 0.4 and 1.19 m in Honolulu by 2050 and 2100, respectively, and the intermediate-high reaches 0.57 and 1.93 m in Honolulu by 2050 and 2100, respectively. The intermediate scenario is of primary focus in this study to support general adaptation planning. However, we suggest the use of more extreme SLR projections (i.e., intermediate-high, high, and extreme) when designing projects that are highly sensitive to flood impacts such as centralized critical infrastructure with no capacity to accommodate flooding^3^.

## Results

### Results of mapping analysis

Map simulations were produced that illustrate locations in which flooding is expected to occur as the result of direct marine inundation, storm-drain backflow, and GWI due to sea level exceedance of the KT2017, minor, and moderate flood thresholds. The KT2017 flood threshold of 0.35 m relative to the MHHW datum represents sea level elevations reached during king tide events in 2017 that produced record high water levels at the Honolulu tide station^7,8^. As part of flood mapping, the three mechanisms are superimposed in the following order: direct marine inundation, storm-drain backflow, GWI, such that direct marine inundation is the top layer featured. To avoid overcomplicating the mapping product, we report results relating to concurrent flood mechanisms in an associated table (Table 1) as areas and percentages of total inundation.

**Table 1:** Multi-Mechanism Flooding Extent. Areas of single and multi-mechanism flooding within the study area encompassing Honolulu’s primary urban center consisting of direct marine inundation (MI), storm-drain backflow (DBF), and groundwater inundation (GWI) at four flood thresholds: KT2017, minor, moderate, and major flood thresholds^5^.

Flood Type	GWI Only		MI Only		DBF Only		Double Mechanism GWI and MI		Double Mechanism GWI and DBF		Double Mechanism MI and DBF		Triple Mechanism GWI, MI, DBF		
	Area Inundated (km^2^)	% of Total	Area Inundated (km^2^)	% of Total	Area Inundated (km^2^)	% of Total	Area Inundated (km^2^)	% of Total	Area Inundated (km^2^)	% of Total	Area Inundated (km^2^)	% of Total	Area Inundated (km^2^)	% of Total	Total Area Inundated (km^2^)
KT2017	0.19	26.33	0.02	2.39	0.00	0.40	0.31	41.98	0.07	10.10	0.00	0.65	0.13	18.15	0.73
Minor	0.24	23.32	0.03	2.66	0.01	0.84	0.38	36.65	0.17	16.14	0.01	0.95	0.20	19.43	1.04
Moderate	0.63	24.74	0.06	2.31	0.10	3.74	0.50	19.56	0.19	7.52	0.08	3.17	0.99	38.95	2.54
Major	1.19	15.19	0.28	3.61	0.04	0.55	1.37	17.55	0.18	2.35	0.47	6.05	4.27	54.71	7.81

#### Preliminary validation using the kt2017 flood simulation

**Figure 3:**
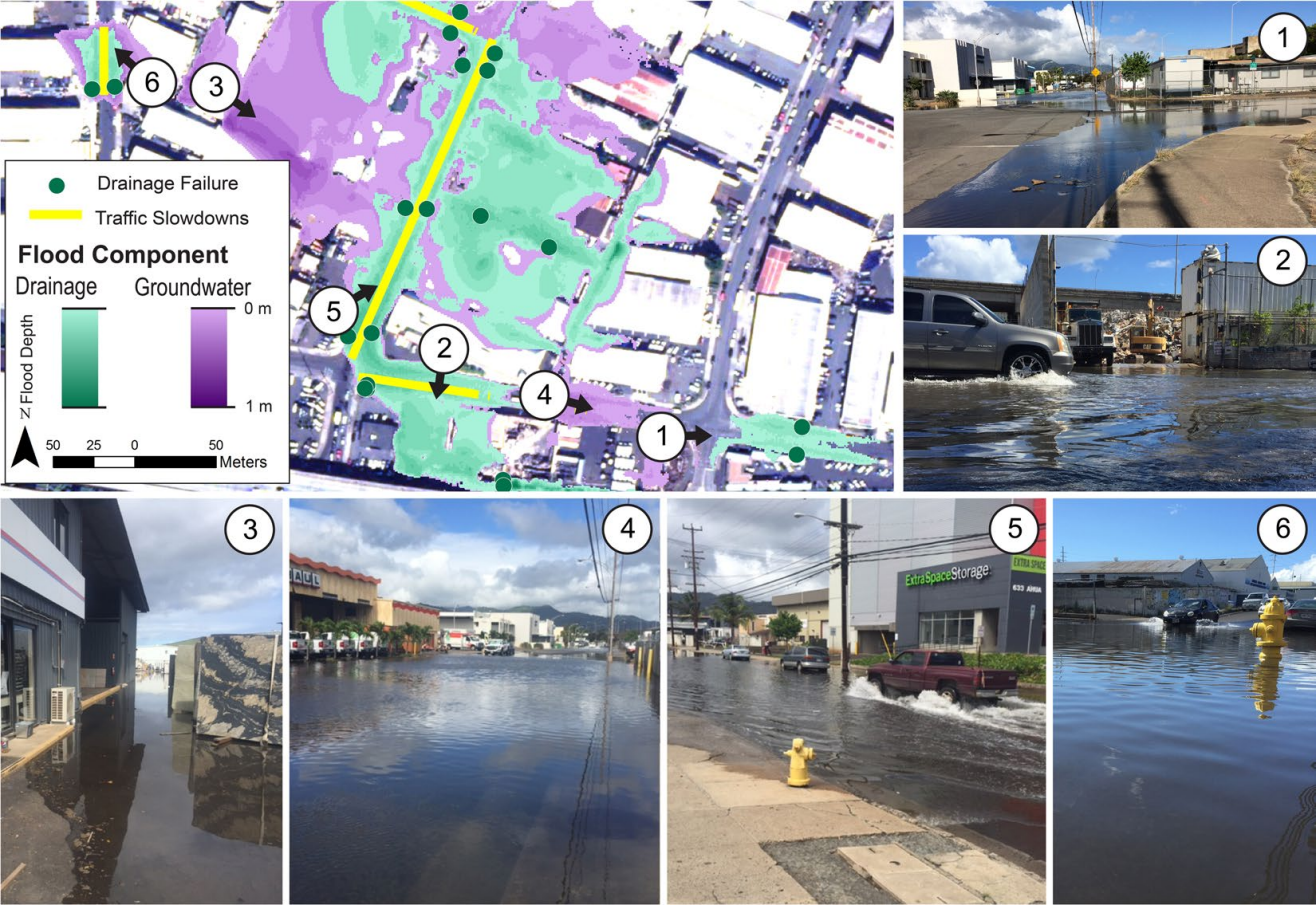
Multi-Mechanism Flooding Considering the KT2017 Flood Threshold. Simulated flood mechanisms in the Māpunapuna industrial area considering the KT2017 flood threshold (0.35 m MHHW). Observations during 2017 king tide events, in which the subject threshold was reached, are shown and locations identified by number on the associated flood simulation. In simulations and observations of this area, only the storm-drain backflow and GWI mechanisms are featured; direct marine inundation is impeded by surface topography and thus not observed or identified in simulations.

The simulation of multi-mechanism flooding considering the KT2017 threshold was compared to images taken during the 2017 king tide event to qualitatively field validate the results (i.e., ground truthing) (Fig. 3). Storm-drains characterized in the simulation as experiencing failure were validated through visual observation. Validation of the GWI simulation was undertaken as part of an associated study that verified the presence of groundwater at simulated locations using geochemical tracers^32^. Simulations have been preliminarily verified as described, however, future studies would benefit from quantitative validations using techniques such as aerial photogrammetry and verification of flood depths produced during simulated tide stages.

#### Flood simulation considering minor and moderate thresholds

**Figure 4:**
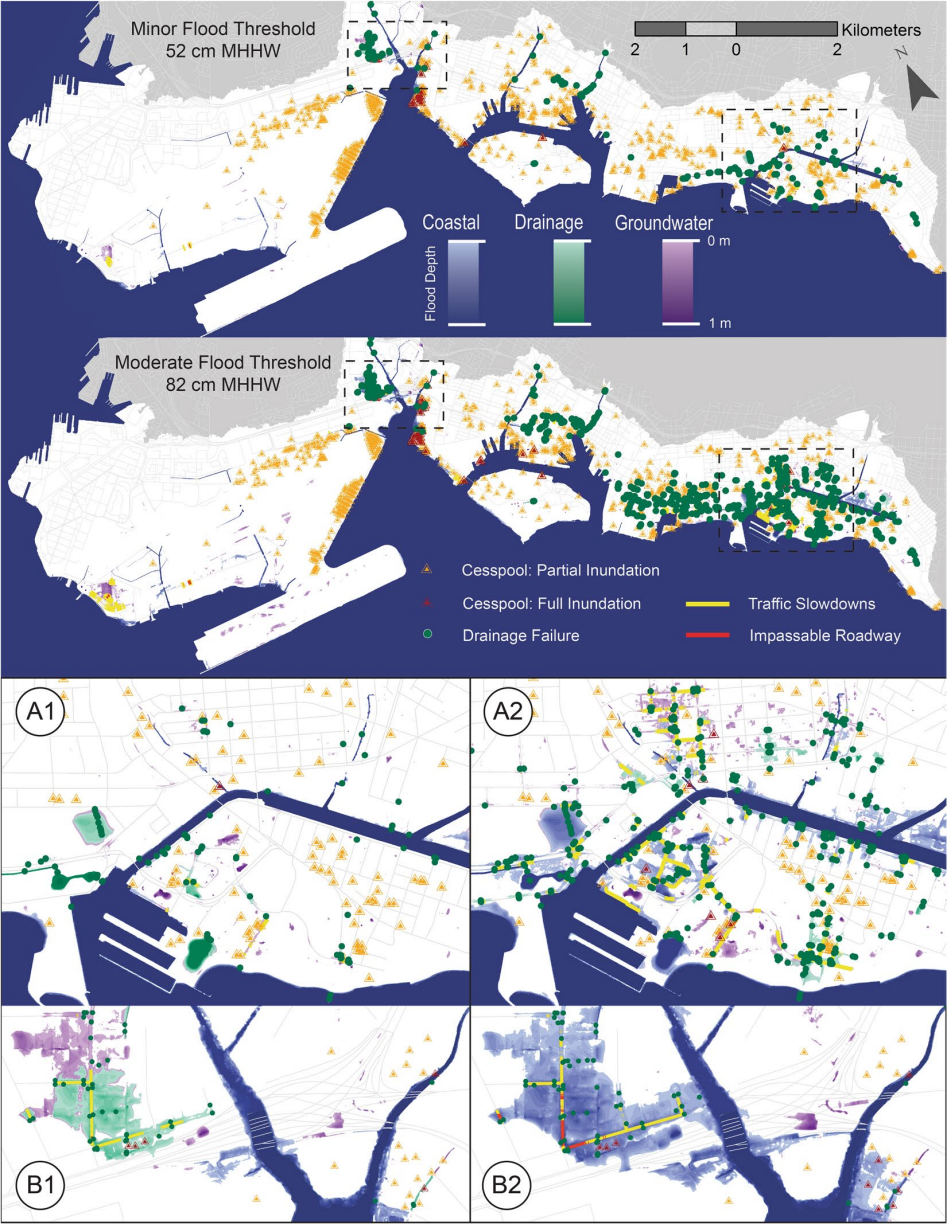
Multi-Mechanism Flooding and Infrastructure Failure. Upper figures show flooding and infrastructure failure across the primary urban center considering minor and moderate flood thresholds. The bottom two subsets show the Waikīkī (A1 and A2) and the Māpunapuna industrial area (B1 and B2). A1 and B1 illustrate flooding at the minor threshold and A2 and B2 illustrate flooding at the moderate threshold.

The minor and moderate thresholds represent elevations of 0.52 m and 0.82 m relative to the MHHW datum, respectively (Fig. 4). Maps were not produced considering the major flood threshold (1.19 m MHHW) since the intent is to focus attention on near term scenarios, although calculations of flooded area and infrastructure failure were completed considering all four thresholds (Tables 1 and 2).

**Table 2:** Analysis of Failed Infrastructure. Length of roadway (km) likely to feature dangerous or impassable driving conditions, number of gravity-flow drainage inlets (out of 6770) likely to fail and/or act as conduits for additional flooding, and number of active cesspools (out of 691) likely to be non-functional at filtering effluent and/or be fully flooded to the ground surface.

Flood Threshold	Traffic Slowdowns (km)	Roadway Impassable (km)	Failed Storm Drains	Non-Functional Cesspools	Fully Flooded Cesspools
KT2017	0.61	0.00	110	619	11
Minor	1.33	0.03	200	620	18
Moderate	9.19	0.40	860	629	38
Major	53.88	4.66	2340	633	112

Overall, multi-mechanism flood simulations and associated damage analyses indicate that flood threats multiply as sea level rises. For example, the percentage of flooded area identified as triple-mechanism flooding increases from 18% at the KT2017 threshold to nearly 55% upon reaching the major threshold. Impacts to roadway, storm-drains, and cesspools also multiply with increasing sea level, apart from non-functional cesspools. The number of non-functional cesspools remains nearly stationary as almost all active cesspools (nearly 90%) are simulated as non-functional considering the lowest flood threshold, illustrating that values are currently near saturation. In the following sections, focus is given to specific flood thresholds regarding multi-mechanism flooding extents and infrastructure impacts.

#### Minor flood threshold

Simulated flooding considering the minor flood threshold indicates that approximately 73% of the flooded area is produced by multi-mechanism flooding by two or more flood types. Of the three mechanisms, GWI is shown to cover the largest area as single-mechanism flooding and by combinations of double and triple-mechanism flooding. Multi-mechanism flooding by GWI and direct marine inundation is shown to inundate approximately 37% of the total flooded area, more than 23% is shown to be inundated solely by groundwater, and approximately 16% of the area is shown to be inundated by combined GWI and storm-drain backflow. The area flooded by single-mechanism direct marine inundation accounts for less than 3% of the total flooded area.

Infrastructure failure featured at the minor flood threshold includes 200 locations where drainage inlets lose all capacity for drainage and begin acting as conduits for flooding, which represents near doubling relative to the KT2017 simulation. Simulated roadway impacts are concentrated mainly in the Waikīkī and Māpunapuna areas and feature traffic slowdowns along 1.3 km of roadway, spread across 22 separate roadway segments and three intersections. Simulated cesspool failure features 620 cesspools characterized as non-functional. Of these, 18 are identified as flooded to the ground surface and the remaining 602 are identified as partially inundated.

#### Moderate flood threshold

Simulated flooding considering the moderate threshold indicates a doubling of flooded area relative to the minor threshold. Calculation of triple-mechanism flooding indicates that the corresponding flood percentage also approximately doubles relative to that of the minor threshold, representing nearly 39% of the total flooded area. Calculation of area flooded by GWI indicates flooding of similar proportion relative to the minor threshold, representing nearly 25% of the total flooded area. Similar to the minor threshold, single-mechanism direct marine inundation remains at less than 3%.

Upon reaching the moderate flood threshold, simulated infrastructure failure is significantly magnified. The number of failed storm-drains increases to 860, a fourfold jump from the minor threshold; the length of dangerous roadway conditions increases to 9.19 km, a nearly seven-fold jump from the minor threshold; and cesspools flooded to the ground surface more than double. Compared to the minor threshold, there is a small increase in failed cesspools to 629 (a number that is already alarmingly high) in which 38 are shown to be fully flooded.

#### Major flood threshold

While the focus of this study is on near term flood scenarios, calculation of impacts represented by the major flood threshold is included to illustrate the trajectory of expected flooding and infrastructure failure. Calculations illustrate two main findings; critical infrastructure and flood area multiply relative to the moderate threshold, and more than half of the total area flooded is identified as resulting from triple-mechanism flooding. Thus, it is likely that an increasing level of adaptation effort will be required if infrastructure is to remain at current elevations.

We acknowledge that this assessment does not represent a full accounting of infrastructure that will be impacted by considered flood mechanisms. To maximize efficiency of design, planning, and workflow, it will likely be important for agencies to conduct their own assessments considering assets they manage and further, to integrate their results with the results of other agencies and utilities.

### Frequency analysis

Frequencies at which sea levels are projected to exceed the KT2017, minor, and moderate flood thresholds are shown, which illustrate the average and maximum number of exceedance days per year for each decade (Table 3).

**Table 3:** Flood Threshold Exceedance Frequencies. Exceedance frequency of the KT2017, minor, and moderate flood thresholds in average and maximum days per year calculated for each decade considering NOAA’s intermediate-low, intermediate, and intermediate-high SLR scenarios^5^.

KT2017	2020		2030		2040		2050		2060		2070		2080	
	Avg	Max	Avg	Max	Avg	Max	Avg	Max	Avg	Max	Avg	Max	Avg	Max
Intermediate-Low	6	18	11	31	49	98	86	168	177	249	221	304	297	340
Intermediate	16	41	42	101	181	274	293	355	361	365	365	365	365	365
Intermediate-High	42	96	126	241	320	362	363	365	365	365	365	365	365	365
**Minor**	Avg	Max	Avg	Max	Avg	Max	Avg	Max	Avg	Max	Avg	Max	Avg	Max
Intermediate-Low	0	0	0	0	1	5	4	15	18	42	35	84	85	146
Intermediate	0	1	1	5	21	57	95	205	279	341	352	365	365	365
Intermediate-High	1	5	10	38	138	255	311	364	365	365	365	365	365	365
**Moderate**	Avg	Max	Avg	Max	Avg	Max	Avg	Max	Avg	Max	Avg	Max	Avg	Max
Intermediate-Low	0	0	0	0	0	0	0	0	0	0	0	0	0	0
Intermediate	0	0	0	0	0	0	0	1	8	25	87	203	279	342
Intermediate-High	0	0	0	0	0	3	33	115	260	349	361	365	365	365

Exceedance frequency calculations are highly dependent on the SLR projection considered. This is indicated by large ranges in average and maximum exceedance days per year calculated for each decade. Calculations indicate that KT2017 threshold exceedance will likely become a daily event as soon as the 2050 s. Exceedance of the minor threshold is shown to begin by the 2020 s to 2040 s depending on which SLR projection is considered. By the 2040 s, average minor threshold exceedance days per year range from 1 to 138, and by the 2080 s exceedance becomes a daily phenomenon considering the intermediate and intermediate-high scenarios. Exceedance of the moderate threshold does not occur by the 2080 s considering the intermediate-low scenario; however, considering the intermediate and intermediate-high scenarios, exceedance is shown to occur by the 2040 s and 2050 s, respectively.

## Discussion

Over the 20th century, urban expansion along Honolulu’s oceanfront has relied heavily on the support of coastal armoring, land reclamation, and channelized gravity-flow drainage. This is true of many densified urban coastal municipalities including New York, Boston, and others^27,41,42^. Since the rapid urbanization began, sea level has risen to the point that exceptionally high tides produce flooding in low-lying densified areas. Results suggest that during present-day extreme high tides in Honolulu, more than a quarter of such flooding is the sole product of GWI, 18% originates from triple-mechanism flooding, and less than 3% is the sole product of direct marine flooding. As sea level continues to rise, proportions of flooding produced solely by GWI and direct marine flooding remain consistent, while the proportion of triple-mechanism flooding multiplies, representing more than half of the flooded area considering the major flood threshold. These findings highlight the potential for GWI and multi-mechanism flooding to produce major impacts moving forward, which illustrates the need for specific and customary consideration of these mechanisms in adaptive planning.

The minor and moderate flood thresholds considered in this study have not yet been reached locally and represent an additional elevation of 0.17 and 0.47 m, respectively, beyond the 0.35 m threshold. These thresholds are expected to be reached as soon as the 2030 s and 2060 s, respectively considering NOAA’s intermediate scenario and by the 2020 s and 2050 s, respectively considering NOAA’s intermediate-high scenario. Results indicate that as water levels advance from the minor to moderate flood threshold, flooded area and infrastructure impacts escalate markedly. The design of flood management strategies required to mitigate these impacts necessitate site-specific consideration of each mechanism to avoid the potential of being rendered ineffective. Exceedance frequency calculations suggest that municipal planning efforts would currently benefit from consideration of flood simulations such as those presented here. For example, the construction lifespan of an average building is generally 50 years^43^; thus, such a building constructed today would have an intended lifespan beyond the decade in which the minor and moderate flood scenarios are likely to occur.

The assessment of critical infrastructure reinforces the need to consider multi-mechanism flood scenarios in present-day municipal planning owing to the extreme vulnerability of specific components that feature limited capacity to accommodate flooding. To mitigate SLR related impacts, simulations of multi-mechanism flooding such as those featured here can be used to prioritize infrastructure upgrades, ideally as part of normal maintenance schedules.

The combination of results regarding GWI and presently occurring cesspool failure reveals the potential for increasingly prevalent public exposure to wastewater contamination. Simulations of cesspool failure considering the KT2017 flood threshold indicate that nearly 90% of cesspools in the study area are likely inundated during present-day king tide events. This suggests that contamination from inundated cesspools is already pervasive. These findings were reinforced as part of an associated study through the detection of wastewater tracers in floodwaters identified as GWI. It was suggested in that study that proximal and potentially compromised active cesspool sites may be the source of detected wastewater tracers^32^.

In many locations across the U.S., onsite wastewater systems have been upgraded such that significantly less vertical unsaturated space is required to provide effective wastewater treatment. However, results of this study remain relevant to such areas, specifically in cases where vertical unsaturated space required for treatment is completely lacking. Resulting contamination of floodwaters by sewage effluent has been cited as a growing concern due to the increased transport of pathogens known to impact public health and coastal ecosystems^44^.

In addition to contamination by sewage effluent, poor land use and waste management practices (i.e. contaminant spills, leaking underground storage tanks, etc.) have contributed to a high level of contamination in groundwater and nearshore coastal waters in the primary urban center^45–47^. In areas vulnerable to GWI, there is potential for these contaminants to reach the ground surface such that focused efforts will likely be required to limit public contact.

## Summary and Conclusion

In Hawai’i, action has been initiated towards adaptation design and implementation, which in-turn requires the identification of site-specific SLR related vulnerabilities. These efforts are judicious; however, alternate SLR induced flood sources of GWI and storm-drain backflow are often overlooked as part of these efforts. Such oversight has the potential to render flood management efforts increasingly ineffective as sea level continues to rise.

Here we produce a streamlined method of simulating locations and depths of flooding generated by multi-mechanisms SLR induced flooding. The method was produced by combining and adapting elements of previous flood mapping efforts and frequency analyses specific to the study area. Frequencies of annual threshold exceedance are established using the methods of Thompson *et al*. that consider predicted tide and projections of SLR. Flood mechanisms assessed include direct marine flooding, storm-drain backflow and GWI, which have not been explicitly simulated and assessed as a combined suite prior to this study.

Flood simulations are produced considering four flood thresholds; 0.35 m, representing known instances of nuisance flooding, and three others established by Sweet *et al*. expected to produce minor, moderate and major flood impacts in the Honolulu area. With the combined method, near-term scenarios of multi-mechanism flooding are produced that consist of high-resolution, site-specific raster datasets featuring water depth generated by the three flood types. Because the majority of the primary urban center shoreline has been hardened, we do not include consideration of dynamic coastal processes (i.e., coastal erosion, wave run-up, changes in land cover).

To illustrate the utility of the method, we conduct a preliminary damage assessment by superimposing geospatial data that characterize locations of drainage, roadways, and cesspools, on flood simulations. We find that infrastructure failure is already occurring during periods of high tide, as illustrated by backflow of gravity drainage, by traffic slowdowns along submerged roadway, and by partial inundation of active cesspools. Considering the NOAA intermediate SLR scenario, the 2030 s will feature localized stretches of roadway that become impassable to 4-wheel drive vehicles (i.e., emergency response vehicles) and drainage failure that is increasingly widespread. In upcoming decades, infrastructure failure of each type assessed is expected to multiply and the amount of area inundated by multi-mechanism flooding is expected to increase fivefold. Here we illustrate that flood management strategies will require consideration of multi-mechanism flooding in which lack of consideration may render such strategies ineffective and lead to a false sense, by key decision makers, that SLR adaptation has been achieved.

## Methods

### Identification of flood thresholds

Coastal flood severity thresholds have been established by Sweet *et al*. (2018) for forecasting purposes and to ensure public safety; these thresholds vary by location and are generally calibrated to NOAA tide gauge stations^5^. The thresholds are defined as minor, moderate and major to respectively describe disruptive, damaging, and destructive coastal flooding, respectively. Sweet *et al*. define local elevation thresholds as a function of local tide ranges along U.S. coastlines to establish a nationally consistent definition of flooding and impacts for the purpose of quantifying and communicating risk. Flood thresholds calculated for Honolulu are 0.52 m, 0.82 m, and 1.19 m for minor, moderate, and major thresholds, respectively, relative to the local mean higher high water (MHHW) tidal datum.

The NOAA thresholds have not been officially established for the Honolulu tide gauge as they have not yet been reached, and in turn, calibrated relative to flood impacts. However, flood thresholds derived for Honolulu were considered valid as part of the study by Sweet *et al*. since topographic characteristics and tidal ranges are represented by locations in which NOAA thresholds have been derived (i.e., stations in South Florida).

Tidally induced flood impacts are known to occur among exceptionally low-lying regions of Honolulu. An additional flood threshold was defined by Thompson *et al*. for the purpose of evaluating the progressive increase in exceedance of water levels known to produce tidally induced flood impacts in these areas. The study qualitatively established a 0.35 m threshold relative to MHHW by comparing photo documentation of flooding to water levels recorded at the Honolulu Harbor Tide Gauge. Since tide gauge records began in 1905, this threshold has been exceeded on 37 distinct days in which fifteen of those days occurred during the summer of 2017^7^.

### Flood frequency prediction

Methods for determining increases in flood frequencies considering SLR were adopted from the method of Thompson *et al*. that projects frequencies at which a defined flood threshold will be exceeded by sea level over the 21st century. The method uses a hierarchical statistical model that describes annual counts of exceedance days as beta-binomially distributed random variables, which allows for exceedance probability on a given day to vary year-to-year as a function of annual mean sea level and the amplitude of the highest tides. Calculations do not consider SLR related non-stationarity of the local tides. Lack of such consideration likely produces slight underestimations in calculated exceedance frequencies; thus, it would be worthwhile to incorporate consideration as part of future studies.

### Flood mapping

#### DEM construction

To define ground elevations on which water levels can be compared, and thus, depth of flooding evaluated, a digital-elevation model (DEM) was constructed by merging and hydroflattening 2013 NOAA DEM tiles. The tiles were constructed by NOAA using raw LiDAR ground return data points that characterize elevations relative to LMSL^48^.

Hydroflattening was accomplished by characterizing areas in which water returns were likely by applying an arbitrary constant elevation of −1.5 m to all major waterways, water features, and offshore areas. All major waterways are thus assumed to be connected to groundwater. The tiles have 1 m horizontal and 0.15 m vertical resolution, respectively^49^. In our results section, flood scenarios were generated considering mechanisms described below and sea level elevations equal to the 0.35 m threshold established by Thompson *et al*., and the minor, moderate and major flood thresholds established by Sweet *et al*. In the mapping and damage analysis it is understood that more than one flood mechanism can be featured in the same location. In such cases, it is assumed that flooding will remain unless all mechanisms featured in that area are mitigated. For example, if GWI and direct marine flooding are featured in an area, and only the direct marine source is mitigated, GWI would remain.

#### Direct marine inundation

Locations vulnerable to the direct marine source of flooding were identified using a variant of the bathtub approach, which characterizes flood vulnerability by identifying areas within a DEM that host elevations below that of chosen flood thresholds^9,10^. Here we alter the method by excluding flooded areas that lack surficial connection to the coastline as it is inferred that the topography would obstruct direct surficial flow from a marine source. The coastline used to determine surficial connectivity was digitized using DEM elevation contours of the respective simulated flood thresholds. Uncertainty regarding simulations of direct marine flooding is directly associated with the vertical resolution of data used to characterize ground elevations, which is 0.15 m as previously stated. The contribution of groundwater to flooding was not considered in the simulation of direct marine inundation.

#### Storm-drain backflow

Locations vulnerable to flooding from storm-drain backflow were also identified using a variant of the bathtub approach. Here we alter the method by excluding flooded areas that lack surficial connection to drainage infrastructure. Drainage inlet elevations were extracted from the DEM. The uncertainty regarding simulations of direct marine flooding is also directly associated with the vertical resolution of data used to characterize ground and drainage inlet elevations. Geospatial data that characterize locations of drainage inlets were used to identify locations where drainage would facilitate flow from a marine source. The hosting agency has high confidence in the accuracy of the data regarding their location to parcel boundaries such that the data is being used extensively by planners, managers, and operational crews to identify network flows, existing assets, and infrastructure maintenance requirements^50^. Flood areas that did not overlap drainage infrastructure were excluded as it is inferred that flooding would not be facilitated by drainage infrastructure. The contribution of groundwater to flooding was not considered in the simulation of storm-drain backflow. Simulation of storm-drain backflow does not incorporate dynamic effects (i.e., variation in water flow rate as a function of conduit radii). Lack of such consideration likely produces slight overestimations in simulated flood depth and area; thus, it would be worthwhile to incorporate consideration as part of future studies.

#### Groundwater inundation

Simulations of groundwater levels were produced using a 3D numerical model (MODFLOW). Methodologies employed in model construction were adopted from Habel *et al*. and expanded according to Habel *et al*. ^51^. The model simulates steady-state conditions of head considering increases in sea level equal to defined flood thresholds, applied as 0.52, 0.82, and 1.19 m, respectively. Regional studies were used to characterize subsurface hydrogeology of the study area^52–56^.

The model was calibrated using 247 discrete water-level observations obtained from Hawai’i Department of Health Leaky Underground Storage Tank records, and 73 sets of continuous water-level measurements compiled from local hydrogeologic studies. Following calibration, the simulated mean residual water level and root-mean-squared error were 0.04 m and 0.12 m, respectively. Further information regarding groundwater model construction and limitations is included in the Supplementary Material.

Locations vulnerable to GWI were identified by comparing simulated water table elevations to the DEM in which flood depth and depth to groundwater were assessed by evaluating elevation differences between the water table and ground surface (i.e., DEM). Flood depths were calculated in locations where water table elevations exceed ground surface elevations while depths to groundwater were calculated in locations in which ground surface elevations exceed water table elevations. The quadrature sum of the two sources of vertical error associated with GWI simulation, which are LiDAR error and calibrated MODFLOW error, was found to be 0.2 m.

### Damage assessment

To illustrate the utility of flood simulations in performing vulnerability analyses, a damage assessment was conducted. As part of the assessment we quantify critical infrastructure failure considering sea levels equal to respective flood threshold elevations and considering the three flood mechanisms simulated. Here we calculate the following impacts.

The length of roadway featuring dangerous driving conditions produced by flood depth is identified where simulated depth of flooding is greater than 0.15 m along roadways. This water depth is based on the depth in which water would enter the intake systems of small vehicles causing them to stall, thus impeding the flow of traffic^57^.

The length of roadway impassable by 4-wheel drive vehicles is identified where simulated depth of flooding is greater than 0.6 m along roadways. This water depth is based on the depth in which water would enter the intake systems of 4 wheel drive vehicles causing them to stall^57^. Roadways are considered impassable once 4-wheel drive vehicles can no longer pass.

Drainage inlets that have no capacity to transport storm water were identified. These inlets act as conduits for additional flooding by storm-drain backflow. Inlet failure is characterized in locations where the elevation of a drainage inlet is equal to or less than simulated water levels. Drainage inlet elevations were extracted from the DEM.

Cesspools that are non-functional at filtering effluent were identified. Non-functioning cesspools are identified using the methods of Habel *et al*. such that cesspool locations lacking 4.4 m of unsaturated space, required by the State Department of Health, indicate partial inundation of the cesspool and inability of the system to properly treat effluent^58^.

We identify the number of cesspools fully flooded to the ground surface. Fully flooded cesspools were also identified using the methods of Habel *et al*. in which the cesspool locations featuring elevations below that of simulated water levels are identified as being fully submerged.

Geospatial data sets that characterize locations of drainage inlets, roadway, and on-site sewage disposal systems were used to conduct the assessment of infrastructure failure^50^.

## Supplementary Information

Supplementary Information.
