# Supplementary material for: Sea-Level Rise Induced Multi-Mechanism Flooding and Contribution to Urban Infrastructure Failure
Source: Sci Rep. 2020 Mar 2;10:3796. doi: 10.1038/s41598-020-60762-4 (PMC7052155; doi:10.1038/s41598-020-60762-4)
Supplement: Supplementary file 1 — Supplementary Information. [file 41598_2020_60762_MOESM1_ESM.docx]

Supplementary material for

“Sea-Level Rise Induced Multi-Mechanism Flooding and Contribution to Urban Infrastructure Failure”

Shellie Habel,1* Charles H. Fletcher, 1, Tiffany Anderson, 1, Philip Thompson,2

*1) University of Hawai‘i at Mānoa, School of Ocean and Earth Science and Technology, Department of Earth Sciences, POST Building, Suite 701, 1680 East-West Road, Honolulu, HI 96822, USA*

*2) University of Hawai‘i at Mānoa, Sea Level Center, 1000 Pope Road, MSB 317, Honolulu, HI 96822*

** Corresponding author. E-mail: skey@hawaii.edu; Phone: (808) 286-2586*

**Supplementary information on the geohydrology of the study area**

The study area is situated to the southwest of the Ko’olau volcanic range and atop a geologic unit referred to as caprock. The unit is composed of a combination of post-erosional volcanics, alluvial debris, and carbonate reef and lagoonal deposits that formed during Pleistocene sea level variations^1–3^, and lies atop basalt flanks of the Ko‘olau Volcano.

Groundwater within the study area occurs mainly as a freshwater lens located in the basalt aquifer, and secondarily in the caprock aquifer^4^. The lens floats atop saltwater due to density differences between salt and freshwater and with an interface approximated by the Ghyben-Herzberg principle^5^. Groundwater migrates down-gradient from areas of recharge to areas of coastal discharge ^6^ in which flow is partially obstructed owing to the lower permeability of caprock relative to basalt^7^. Groundwater levels in the caprock aquifer are influenced by various phenomena including rainfall and marine oscillations ^8–11^. Well withdrawal from the caprock aquifer is minimal owing to the lack of potable water, and is mainly employed for use in cooling towers and for small-scale irrigation.

**Supplementary information on groundwater-level simulation**

The following data and methods were adopted from a study by Habel et al. (2017) and modified in accordance with Habel et al. (2019) unless otherwise noted^12,13^.

*Groundwater-Level Data*

Groundwater-level observations used in 3D model calibration and simulation of tidal influence included 247 discrete water-level observations obtained from Hawai'i Department of Health Leaky Underground Storage Tank records^14^, and 73 sets of continuous water-level observations compiled from local hydrologic studies. Discrete groundwater-level observations were corrected for tidal influence and anomalous sea-surface height using the methods of Habel et al (2017).

*3D Model Construction*

The method employs MODFLOW 2005^15^, a 3D finite-difference flow model, to simulate steady-state conditions of the water table considering various magnitudes of sea level rise. Subsurface hydrogeologic conditions were simulated based on conditions determined in regional studies^1,16–19^. The model consists of 48,483 active 100-m uniform grid cells and three layers that represent unconsolidated caprock (model layer 1), consolidated caprock (model layer 2), and basalt (model layer 3) hydrogeological units. The top of model layer 1 (unconsolidated caprock unit) is defined by mosaicked 2013 NOAA LiDAR topography data and 2013 US Army Corps of Engineers LiDAR bathymetry data^20^ and given a specified thickness of 10 m based on the approximate depth in which consolidated caprock material has been encountered in drilling studies^1,16,17^. Model layer 2 (consolidated caprock unit) extends from the base of layer 1 to the uppermost extent of model layer 3 (basalt unit), which was defined using elevation data that represents the uppermost extent of the basalt aquifer^19^. Simulation of groundwater flow in the basalt aquifer is beyond the scope of this study; thus, model layer 3 is represented by a thin unit that extends an arbitrary 1 m below the top of the layer and was included to simulate flow from the basalt into the caprock aquifer represented by model layers 1 and 2.

The inland boundary of the model is defined by the 0 m elevation contour representing the uppermost extent of the basalt aquifer^19^. The seaward boundary is defined by the 200-m depth contour of 2013 US Army Corps of Engineers LiDAR bathymetry data^20^. With the exception of the inland boundary of layer 3, the model domain is bounded on the sides and base by no-flow boundaries; the upper-boundary is a specified recharge boundary. The inland lateral boundary of model layer 3 is a specified head boundary that simulates flow from the basalt unit to the upper layers, in which specified-head values were based on simulations of confined groundwater flow in southern O'ahu^19^. Locations and withdrawal rates of pumping well locations available from the State Commission on Water Resource Management were adopted from existing groundwater-flow models representative of the Honolulu aquifer^19^; only wells pumping from the caprock aquifer were considered. Withdrawal rates were defined using the arithmetic mean of respective pumping rates from 1996 to 2005. Recharge data were adopted from a mean annual water-budget model representing the Island of O‘ahu, Hawai‘i^21^ that simulates hydrological processes including rainfall, fog interception, evapotranspiration, direct runoff, irrigation and return flow from septic systems. Seaward of the 0-m land-surface elevation contour, conditions of mean sea level were simulated using a specified general-head boundary at the ocean bottom with a conductance of 10 m2 d−1 and a general-head elevation of 0 m .

*Model Calibration*

Model calibration was accomplished using discrete and continuous water-level measurements described previously. All groundwater-level observations were used for 3D model calibration, unlike the methods of Habel et al. (2019) in which groundwater-level observation datasets were subsampled for use in cross-validation analysis. The nonlinear inverse modeling utility, PEST, using Tikhonov preferred homogeneous regularization was employed to estimate hydraulic-conductivity values for model layer 1 representing the unconsolidated caprock unit ^22^; pilot points were established on 500-m grid across the study area, totaling 361 points. All post-calibration values applied to the unconsolidated caprock unit were within the range of values previously observed for the study area, ranging from 0.001 to 854 m/d ^1^ with an average value of 117.4 m/d and standard deviation of 271.0 m/d, which are comparable to values calculated in Habel et al. (2019) of 135.2 m/d and 288.5 m/d, respectively. Manual iterative adjustment was employed to estimate the hydraulic-conductivity value for layer 2, which was set to 1 m d^−1^. For layer 3 the hydraulic conductivity was set to 600 m d^−1^ based on values employed in modeling studies that simulate local basalt aquifers ^19,23^. Vertical anisotropy (Kh/Kv) was set to 3.0 for all layers.

The simulated mean residual water level and root-mean-squared error were 0.04 m and 0.12 m, respectively, following model calibration.

*Simulation of SLR*

Increases in mean sea level were simulated by setting the general head to a value equal to that of the simulated sea level increase. For example, an increase in mean sea level of 1 m is simulated by setting the value of general head to 1 m. Further, the landward extent of the general head boundary was re-evaluated using the land-surface elevation contour equal to that of the simulated sea level increase.

*Limitations of the Groundwater Model*

Limitations of the modeling methodology have been summarized in Habel et al (2017) in which main limitations include:

- The model is steady-state and thus does not assess time-dependent processes (i.e., variations in boundary flows, recharge, pumping rates, and groundwater storage, aperiodic short-term changes in sea level by phenomena such as tsunamis, storm-surges, etc.);
- MODFLOW-2005 assumes uniform density of water, and thus does not assess the influence of density-driven fluid flow including mixed seawater and freshwater flows;
- The model does not consider the following: flow that occurs in the unsaturated zone, surface-water flow, evaporation from surface-water sources, ponding or routing of waters that occurs once groundwater breaches the ground surface, dynamic changes in landscape (i.e., erosion);

*Calculation of Tidal Influence*

Groundwater-level oscillations are attenuated relative to oscillations in ocean water levels, increasing in temporal lag and decreasing in amplitude as they propagate inland. The influence of ocean oscillations was quantified for each set of continuous observations. Temporal lag was evaluated by cross correlating tidal signals at the NOAA Honolulu Tide station with those observed in the groundwater data^24^. Tidal efficiency was calculated using linear least-squares regression of lag-corrected groundwater time series to tidal-signal data.

*Application of Tidal Influence to Simulated Groundwater Levels*

Analytical solutions representing tidal influence on groundwater were evaluated for six subzones by performing regression analyses that assign tidal efficiency as an exponential function of distance from the coastline. Subzones represent the eastern (Waikiki and surrounding neighborhoods), middle (Iwilei and surrounding neighborhoods), and western (Hickam area) extent of the study area. The western extent was further divided into four subzones to represent unique patterns of observed tidal efficiencies that correlated with distinct geologic strata that comprise the subsurface geology (i.e., fill, beach deposits, lagoon and reef deposits, and Honolulu Volcanics)^25^. Tidal influence is expressed as increases in piezometric head considering the tidal half amplitude (h0) by the following analytical solutions in which h(x) is the increase in piezometric head (m), and x is the distance from the shoreline (m):

h(x)/h0 = e−0.002x (eastern)

= e−0.005x (middle)

= 0.55e−0.0007x (western-fill)

= 0.43e−0.001x (western-beach deposits)

= 0.44e−0.0004x (western-lagoon and reef deposits)

= 0.43e−0.0002x (western-Honolulu Volcanics)

The computed tidal efficiencies are considered reasonable and consistent with the hydraulic properties based on the estimated diffusivities for these units.

The six analytical solutions were applied to a raster grid within respective subzone boundaries as a function of the distance of each grid cell to the simulated coastline. Raster values representing tidal efficiency were calculated by setting h0 to 0.33 (MHHW tide elevation relative to the MSL datum in meters). These values were summed with water-table raster data from the groundwater model output to generate the tidally influenced water-table height considering the MHHW tide stage.

**References**

1. Finstick, S. A. *Subsurface geology and hydrogeology of downtown Honolulu with engineering and environmental implications*. (Water Resources Research Center, University of Hawaii at Manoa, 1996).

2. Oki, D. S., Souza, W. R., Bolke, E. L. & Bauer, G. R. Numerical analysis of the hydrogeologic controls in a layered coastal aquifer system, Oahu, Hawaii, USA. *Hydrogeol. J.* **6**, 243–263 (1998).

3. Stearns, H. T. & Vaksvik, K. N. *Geology and ground-water resources of the island of Oahu, Hawaii*. (Maui Publishing Company, Limited, 1935).

4. Gingerich, S. B. & Oki, D. S. *Ground water in Hawaii Fact Sheet 126-00*. (US Geological Survey, 2000).

5. Macdonald, G. A., Abbott, A. T. & Peterson, F. L. *Volcanoes in the sea: the geology of Hawaii*. (University of Hawaii Press, 1983).

6. Souza, W. R. & Voss, C. I. Analysis of an anisotropic coastal aquifer system using variable-density flow and solute transport simulation. *J. Hydrol.* **92**, 17–41 (1987).

7. Stearns, H. T. Pleistocene shore lines on the islands of Oahu and Maui, Hawaii. *Geol. Soc. Am. Bull.* **46**, 1927–1956 (1935).

8. Gonneea, M. E., Mulligan, A. E. & Charette, M. A. Climate-driven sea level anomalies modulate coastal groundwater dynamics and discharge. *Geophys. Res. Lett.* **40**, 2701–2706 (2013).

9. Ponte, R. M. Understanding the relation between wind-and pressure-driven sea level variability. *J. Geophys. Res. Ocean.* **99**, 8033–8039 (1994).

10. Wu, J., Zhang, R. & Yang, J. Analysis of rainfall-recharge relationships. *J. Hydrol.* **177**, 143–160 (1996).

11. Yin, B. *et al.* Numerical study of the influence of waves and tide-surge interaction on tide-surges in the Bohai Sea. *Chinese J. Oceanol. Limnol.* **19**, 97–102 (2001).

12. Habel, S., Fletcher, C. H., Rotzoll, K. & El-Kadi, A. I. Development of a model to simulate groundwater inundation induced by sea-level rise and high tides in Honolulu, Hawaii. *Water Res.* **114**, 122–134 (2017).

13. Habel, S., Fletcher, C. H., Rotzoll, K., El-Kadi, A. I. & Oki, D. S. Comparison of a simple hydrostatic and a data-intensive 3D numerical modeling method of simulating sea-level rise induced groundwater inundation for Honolulu, Hawai’i, USA. *Environ. Res. Commun.* **1**, 041005 (2019).

14. State of Hawaii Department of Health. Environmental Health Warehouse. (2019). Available at: http://eha-web.doh.hawaii.gov/ehw/.

15. Harbaugh, A. W. *MODFLOW-2005, the US Geological Survey modular ground-water model: the ground-water flow process*. (US Geological Survey Reston, VA, USA, 2005).

16. Ferrall, C. C. Subsurface geology of Waikiki, Moiliili and Kakaako with engineering application. (University of Hawaii, Manoa, 1976).

17. Munro, K. The subsurface geology of Pearl Harbor with engineering application. (University of Hawaii, Geology and Geophysics, 1981).

18. Oki, D. S. *Numerical simulation of the effects of low-permeability valley-fill barriers and the redistribution of ground-water withdrawals in the Pearl Harbor area, Oahu, Hawaii. Scientific Investigations Report 2005-5253*. (US Geological Survey, 2005).

19. Rotzoll, K. & El-Kadi, A. I. *Numerical ground-water flow simulation for Red Hill fuel storage facilities, NAVFAC Pacific, Oahu, Hawaii*. *University of Hawaii & Water Resources Research Center, prepared for TEC Inc., Honolulu, Hawaii* (2007).

20. National Oceanic and Atmospheric Administration (NOAA). United States Interagency Elevation Inventory. *2017* Available at: https://coast.noaa.gov/inventory/. (Accessed: 3rd July 2017)

21. Engott, J. A., Johnson, A. G., Bassiouni, M., Izuka, S. K. & Rotzoll, K. *Spatially distributed groundwater recharge for 2010 land cover estimated using a water-budget model for the Island of O‘ahu, Hawai‘i*. *Scientific Investigations Report* (2017). doi:10.3133/sir20155010

22. Doherty, J. E. & Hunt, R. J. *Approaches to highly parameterized inversion: a guide to using PEST for groundwater-model calibration*. (US Department of the Interior, US Geological Survey, 2010).

23. Izuka, S. K. *et al.* *Volcanic aquifers of Hawai‘i—Hydrogeology, water budgets, and conceptual models*. *Scientific Investigations Report* (2018). doi:10.3133/sir20155164

24. National Oceanic and Atmospheric Administration (NOAA). Tide Station 1612340 Station Info. (2017). Available at: http://tidesandcurrents.noaa.gov/inventory.html?id=1612340. (Accessed: 20th October 2016)

25. Sherrod, D. R., Sinton, J. M., Watkins, S. E. & and Brunt, K. M. *Geologic Map of the State of Hawai`i: U.S. Geological Survey Open-File Report 2007-1089*. *US geological survey open-file report* **1089**, (2007).
